# Subendocardial Late Gadolinium Enhancement Without Troponin Elevation: A Case of Apical Hypertrophic Cardiomyopathy With Myocardial Involvement

**DOI:** 10.7759/cureus.98035

**Published:** 2025-11-28

**Authors:** David Martinez Juarez, Omar Gomez-Monterrosas, Angel Antonio Perez Mendoza, Francisco Zamora Rosales

**Affiliations:** 1 Department of Radiology/Cadiovascular Imaging, Christus Muguerza Hospital Betania, Puebla, MEX; 2 Department of Cardiology, Hospital Angeles Puebla, Puebla, MEX; 3 Department of Investigation, Christus Muguerza Hospital Betania, Puebla, MEX

**Keywords:** apical hypertrophic cardiomyopathy, case report, chest pain, myocardial involvement, yamaguchi syndrome

## Abstract

Apical hypertrophic cardiomyopathy (AHCM), also known as Yamaguchi syndrome, is a rare variant of hypertrophic cardiomyopathy (HCM) characterized by localized thickening of the left ventricular apex. Clinically, it may mimic acute coronary syndrome (ACS), myocarditis, or pericarditis. Cardiac magnetic resonance (CMR) allows the identification of myocardial involvement even in the absence of elevated serum biomarkers.

A 39-year-old Hispanic man presented with a four-day history of oppressive chest pain radiating to the left arm, associated with dyspnea, fever, and dysuria. On admission, ECG demonstrated deep T-wave inversions; initial risk stratification (a thrombolysis in myocardial infarction (TIMI) score of 3 and a history, ECG, age, risk factors, and troponin (HEART) score of 4) indicated intermediate probability of ACS. hs-cTn remained negative; however, laboratory tests showed leukocytosis with neutrophilia, elevated inflammatory markers, and a positive urine culture for *Escherichia coli*. The transthoracic echocardiogram (TTE) showed apical hypokinesia and hypertrophy, and coronary CT angiography (CCTA) excluded obstructive disease, while CMR confirmed apical hypertrophy with evidence of myocardial involvement. The patient improved with targeted antibiotic and medical therapy and was discharged with outpatient cardiology follow-up for risk stratification and consideration of implantable cardioverter-defibrillators (ICD); however, it was deferred as no advanced atrioventricular (AV) block, syncope, or ventricular arrhythmias were documented.

AHCM is infrequent in the Hispanic population and may present with chest pain and intermediate risk scores for ACS despite negative troponins. Myocardial involvement, characterized by CMR, helps identify the etiology of chest pain and differentiate ischemic from nonischemic injury, even when serum biomarkers are normal or inconclusive, which in our case was due to the intrinsic microvascular dysfunction of AHCM, likely aggravated by the systemic inflammatory response syndrome (SIRS) triggered by a urinary tract infection.

## Introduction

Acute chest pain remains one of the leading causes of emergency department visits [1], and clinical management usually focuses on ruling out acute coronary syndrome (ACS). However, a subgroup of patients, such as those with AHCM, present with chest pain, electrocardiographic abnormalities, intermediate risk scores, and sometimes negative cardiac biomarkers. In these cases, the risk of misdiagnosis and unnecessary therapies is considerable [2,3].

Apical hypertrophic cardiomyopathy (AHCM) (Yamaguchi syndrome) poses a particular diagnostic challenge under these circumstances. First described in Japanese populations in 1976, its prevalence is substantially higher in Asia [4], whereas it accounts for less than 11% of HCM cases in Western cohorts and is particularly rare among Hispanic patients [5,6].

Clinically, AHCM may present with chest pain, dyspnea, palpitations, or syncope [7]. Electrocardiographic features such as giant negative T waves, transient ST elevation, or axis deviation are typical but often mimic ACS, acute pericarditis, or myocarditis, thereby complicating the initial diagnosis [8].

CMR is the reference standard for myocardial tissue characterization [9], enabling precise identification of apical hypertrophy. In addition, CMR findings carry prognostic implications, including arrhythmic risk stratification and prediction of long-term outcomes [10].

The concept of myocardial involvement refers to tissue-level abnormalities of the myocardium detectable by cardiac magnetic resonance (CMR) (such as fibrosis, edema, or increased extracellular volume), even in the absence of elevated serum biomarkers. This framework, highlighted in position statements of the European Society of Cardiology (ESC), is crucial to recognizing myocardial involvement in structural cardiomyopathies and systemic inflammatory conditions [11].

## Case presentation

A 39-year-old Hispanic male patient with a history of recently diagnosed systemic hypertension under treatment with enalapril 10 mg daily and dyslipidemia was managed with atorvastatin 20 mg daily. He reported tobacco use and occasional alcohol consumption. There was no family history of cardiomyopathy or sudden cardiac death.

Four days prior to admission, he developed oppressive chest pain radiating to the left arm, accompanied by mild dyspnea. The pain was intermittent, non-exertional, and worsened with deep inspiration. He denied diaphoresis or syncope. Concomitantly, he reported unquantified fever and dysuria for a few days.

**Figure 1:**
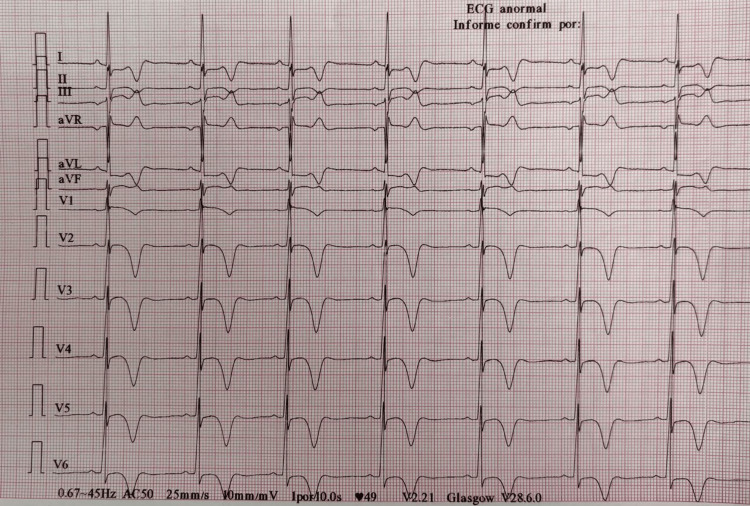
Admission electrocardiogram aVL: augmented vector left Electrocardiogram showing sinus bradycardia, prolonged QTc (475 ms), voltage criteria for left ventricular hypertrophy, and deep symmetric T-wave inversions in precordial (V2-V6) and lateral leads (I, aVL)

He self-medicated with enalapril, acetylsalicylic acid, and paracetamol without clinical improvement and presented to the emergency department. On admission, he was alert, anxious, and afebrile, with persistent chest pain and dysuria. Vital signs were as follows: blood pressure of 106/74 mmHg, heart rate of 55 bpm, respiratory rate of 20 rpm, temperature 36.5°C, and oxygen saturation of 91% on room air. Physical examination showed a symmetric chest, no jugular venous distension, and normal heart sounds without murmurs. The initial ECG (Figure 1) revealed sinus bradycardia, prolonged QTc (475 ms), criteria for left ventricular hypertrophy, and predominantly deep, symmetric T-wave inversions in precordial and lateral leads.

Initial risk stratification yielded a thrombolysis in myocardial infarction (TIMI) score of 3 and a history, ECG, age, risk factors, and troponin (HEART) score of 4, given the initial suspicion of non-ST-elevation acute coronary syndrome (NSTE-ACS).

Transthoracic echocardiography (TTE) showed apical hypertrophy with hypokinesia of the apical segment, grade II diastolic dysfunction of the left ventricle, pericardial hyper-reflectivity, and preserved ejection fraction. The left ventricular outflow tract (LVOT) gradient was within normal limits. He was initially managed with treatment for NSTE-ACS with acetylsalicylic acid, isosorbide, and metoprolol, as well as colchicine for suspected pericarditis, which was later discontinued.

Laboratory tests (Table 1) demonstrated a leukocytosis of 15,600/mm³ and neutrophilia of 81.1%; elevated erythrocyte sedimentation rate (ESR) of 35 mm/h (normal 0-15 mm/h), procalcitonin of 1.22 ng/mL (normal <0.05 ng/mL), D-dimer of 2598 ng/mL (normal <500 ng/mL) and C-reactive protein (CRP) of 0.50 mg/dL (normal <0.5 mg/dL). High-sensitivity troponin I of 0.02 ng/mL (normal <0.30 ng/mL) was measured at admission, six hours, and 24 hours later, showing no dynamic changes or elevation above the reference range. The lipid panel was within normal range under statin therapy.

**Table 1:** Laboratory and urine test results during hospitalization and discharge CRP: C-reactive protein; ESR: erythrocyte sedementation rate

Parameter	Admission	Discharge	Reference values
High-sensitivity troponin I (ng/mL)	0.02	0	0.00-0.30
D-dimer (ng/mL)	2598	899	<500
Leukocytes (/mm³)	15,600	8,200	4,000-11,000
Neutrophils (%)	81.1	62	40-70
CRP (mg/dL)	0.50	0.40	<0.5
Procalcitonin (ng/mL)	1.22	1.1	<0.5
ESR (mm/h)	35	–	0-15
Urinalysis			
Bacteria	Abundant	Absent	Absent
Nitrites	Positive	Negative	Negative
Red blood cells	Numerous	Absent	<5/field
Proteins (mg/dL)	75	Absent	Absent
Leukocytes (/field)	25	1	<5

**Figure 2:**
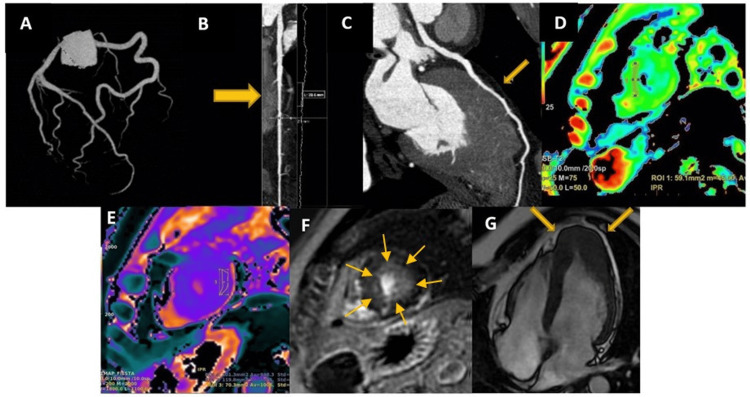
CCTA and CMR CCTA: coronary computed tomography angiography; ECV: external electrical cardioversion; LGE: late gadolinium enhancement; SSFP: steady-state free precession Image A-C: CCTA showing no obstructive coronary disease; a superficial myocardial bridge is seen in the mid-left anterior descending artery (arrow).  Image D: T2 mapping demonstrating increased values in the apical segment (56 ms). Image E: Native T1 mapping elevated (1006-1013 ms), ECV of 30%. Image F: LGE with subendocardial enhancement at the apex (arrow). Image G: Cine SSFP four-chamber view showing apical hypertrophy with a “spade-like” configuration

Coronary computed tomography angiography (CCTA) ruled out obstructive coronary artery disease and demonstrated a superficial myocardial bridge, 20 mm in length and 1.5 mm in depth, in the left anterior descending artery (Figures 2A-2C).

CMR ruled out pericarditis and confirmed apical hypertrophy measuring 17 mm with a “spade-like” configuration. Myocardial involvement was documented with increased native T1 mapping (1013 ms; normal <1000 ms), T2 mapping (56 ms; normal ≤55 ms), and ECV (30%; normal <30%), consistent with inflammation, edema, and fibrosis. Subendocardial LGE at the apical segment showed an atypical pattern for hypertrophic cardiomyopathy (HCM), whereas patchy intramyocardial enhancement in the septal wall displayed a typical HCM distribution, accounting for 11.3% of myocardial volume (Figures 2D-2G). Ejection fraction by CMR was 69%.

During hospitalization, urinary symptoms persisted. Urinalysis revealed leukocyturia of 25/field (normal <5/field), bacteriuria, proteinuria of 75 mg/dL (normal value absent), nitrites positive, and microscopic hematuria. Urine culture demonstrated growth of* Escherichia coli* sensitive to nitrofurantoin; blood cultures were negative. Following targeted antibiotic therapy, inflammatory markers decreased significantly, and symptoms improved progressively (Table 1).

NSTE-ACS, active myocarditis, pericarditis, and infiltrative disease were excluded. The final diagnosis was AHCM with myocardial involvement as the cause of angina. The patient had a favorable clinical course, with progressive resolution of chest pain and no major complications. He was discharged on medical therapy with telmisartan and metoprolol, with outpatient cardiology follow-up for arrhythmic risk stratification. Although CMR demonstrated LGE of 11.3% of the total myocardial mass (a known risk modifier in HCM), the patient did not fulfill the criteria for implantable cardioverter-defibrillator (ICD) placement.

## Discussion

This case illustrates an uncommon clinical scenario in which two distinct mechanisms contributed to myocardial involvement in the setting of AHCM. First, microvascular dysfunction secondary to apical hypertrophy resulted in relative and intermittent ischemia. Myocyte hypertrophy and increased metabolic demand, combined with narrowing of small intramural arteries, favored hypoperfusion in the absence of epicardial coronary disease. This mechanism has been associated with fibrosis, arrhythmias, and adverse long-term outcomes [10].

Second, a systemic inflammatory response syndrome (SIRS) was documented during hospitalization, triggered by a urinary tract infection due to *Escherichia coli*, with leukocytosis, elevated procalcitonin, and D-dimer. Even in the absence of bacteremia, cytokine release (IL-6, TNF-α, IL-1β) may induce endothelial dysfunction, increased capillary permeability, and myocardial edema, which could suggest worsening of myocardial involvement by SIRS. Similar myocardial alterations have been described in systemic inflammatory conditions of autoimmune, autoinflammatory, and infectious origin, such as COVID-19 [11,12].

**Figure 3:**
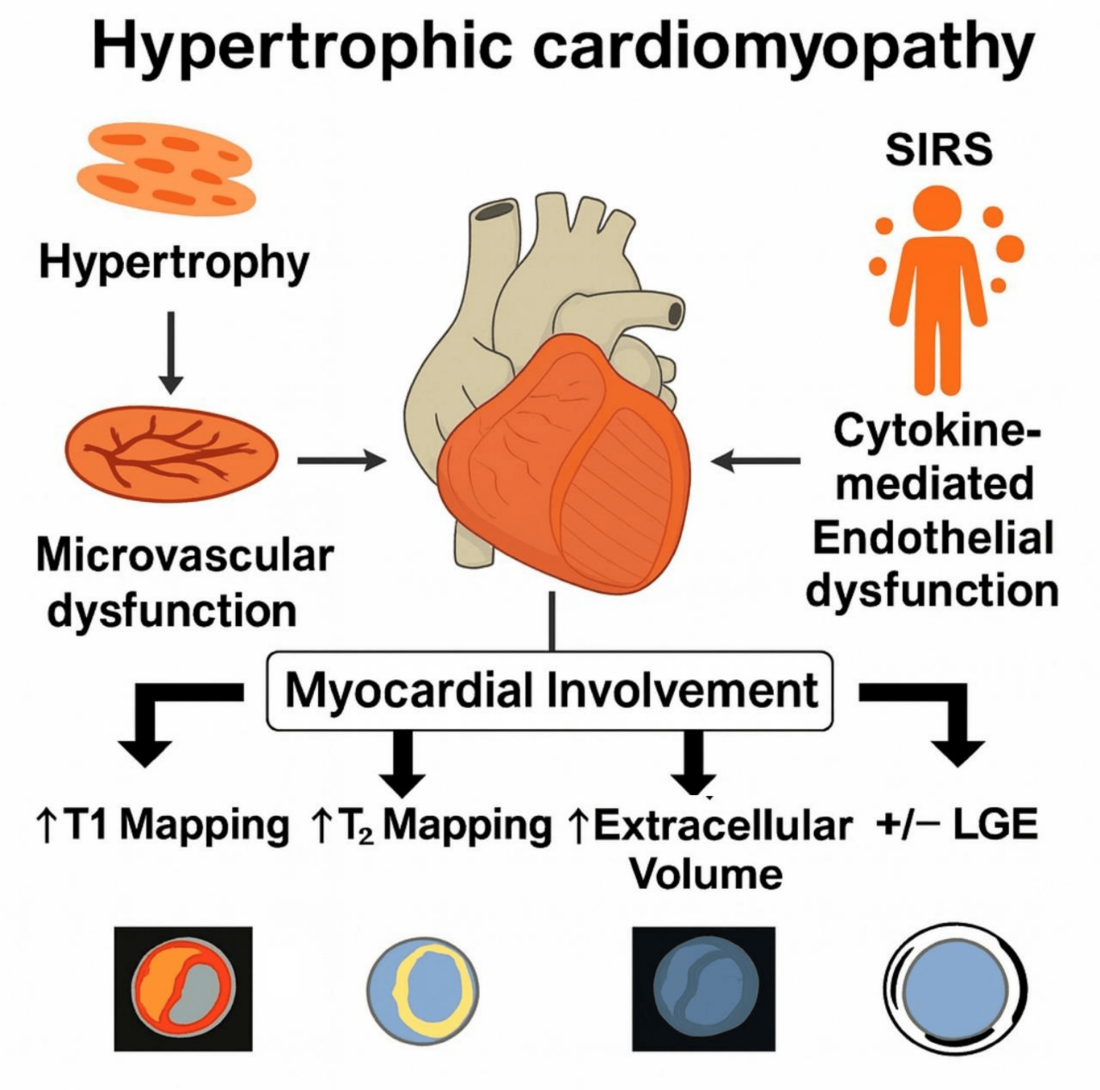
Mechanisms of myocardial involvement in apical hypertrophic cardiomyopathy SIRS: systemic inflammatory response syndrome; ECV: external electrical cardioversion; CMRI: cardiac magnetic resonance imaging Myocardial involvement in apical hypertrophic cardiomyopathy resulting from two converging mechanisms: microvascular dysfunction secondary to apical hypertrophy and cytokine-mediated endothelial dysfunction during SIRS. These processes led to increased T1 mapping, T2 mapping, and ECV on CMRI

The initial differential diagnosis was challenging. The patient presented oppressive chest pain radiating to the left arm, with pleuritic features (pain worsened by inspiration), suggestive of pericarditis. In addition, the TTE showed pericardial hyper-reflectivity, further supporting this suspicion. Electrocardiographic abnormalities (giant negative T waves) and intermediate risk scores (TIMI 3, HEART 4) reinforced the consideration of NSTE-ACS. In accordance with the 2020 ESC guidelines for the management of NSTE-ACS, early risk stratification with clinical scores and biomarkers is essential in patients presenting with acute chest pain. However, both CCTA and CMR excluded these entities and confirmed a structural cardiomyopathy [13]. Missing AHCM during the initial evaluation of chest pain may lead to unnecessary antithrombotic therapy or even unwarranted coronary catheterization.

An additional finding on CCTA was a superficial myocardial bridge in the mid-left anterior descending artery; up to 25% of the population, most cases are clinically silent. Only a minority are associated with dynamic compression and exercise-induced ischemia [14]. In our patient, the bridge was short and shallow, making a direct clinical impact unlikely. Nevertheless, its identification underscores the role of multimodality imaging in excluding obstructive coronary disease and contextualizing concomitant anatomic variants.

Although cardiac biomarkers were negative, CMR demonstrated subendocardial LGE, myocardial edema, and ECV expansion. According to the position statement of the ESC Working Group on Myocardial and Pericardial Diseases, myocardial involvement refers to tissue abnormalities detectable by CMR even in the absence of elevated serum biomarkers [11].

In our patient, CMR revealed a subendocardial LGE at the apical segment (atypical pattern in HCM) and patchy intramyocardial enhancement along the mid-septal wall. These findings highlight the ischemic and arrhythmic vulnerability despite negative troponins [7]. Furthermore, abnormal mapping parameters provide complementary prognostic information: elevated native T1 and ECV reflect diffuse interstitial fibrosis and predict adverse remodeling [15]. Increased T2 values indicate myocardial edema and correlate with acute symptom burden and short-term risk [16,17].

Another relevant aspect in this case was the evaluation of whether the patient met the criteria for ICD (Table 2). Although CMR demonstrated myocardial fibrosis involving 11.3% of the total myocardial mass (a finding recognized as a risk modifier), the patient did not meet the major sudden cardiac death (SCD) risk criteria established by the American Heart Association and the American College of Cardiology (AHA/ACC) [18] (unexplained syncope, non-sustained ventricular tachycardia, family history of SCD, extreme left ventricular hypertrophy (≥30 mm), or apical aneurysm), which ruled out the primary indication for ICD implantation. It is important to emphasize that although LGE has been associated with a progressive increase in arrhythmic risk, its isolated presence does not constitute a formal indication for ICD placement, particularly when its extent is moderate (<15%) [18], as in our patient. Therefore, management focused on close follow-up and symptom control, with periodic reassessment of arrhythmic risk.

**Table 2:** Major imaging risk markers and CMR markers associated with increased arrhythmic risk in hypertrophic cardiomyopathy (AHA/ACC 2020) AHA/ACC: American Heart Association and the American College of Cardiology This table separates the major risk markers that may justify ICD implantation from CMR findings associated with increased arrhythmic risk, which may contribute to risk stratification but do not represent standalone indications according to the 2020 AHA/ACC guideline Source: [18]

Major imaging-based risk markers (may justify ICD)		
Risk marker	AHA/ACC 2020 category	Clinical meaning
Maximum LV wall thickness ≥30 mm (echo or CMR).	Major SCD risk marker (Class IIa).	Strong predictor of SCD.
LV apical aneurysm (CMR or ventriculography).	Major SCD risk marker (Class IIa).	Associated with ventricular arrhythmias and thromboembolism.
Left ventricular ejection fraction (LVEF) ≤50%.	Major SCD risk marker (Class IIa).	Associated with adverse prognosis.
CMR markers associated with increased risk (not stand-alone indications)		
CMR finding	Description	Clinical meaning
Extensive LGE ≥15% of LV mass.	Associated with increased SCD risk.	Helps justify ICD in borderline or intermediate-risk cases.
Typical intramyocardial HCM LGE.	Common fibrosis pattern; contributes to risk estimation.	Prognostic.

## Conclusions

This case highlights the value of CMR in identifying myocardial involvement in AHCM patients presenting with chest pain, with negative troponins and clinical features suggestive of ACS or pericarditis. The coexistence of microvascular dysfunction due to apical hypertrophy and a SIRS response secondary to urinary tract infection amplified myocardial vulnerability, underscoring the importance of a multimodality approach to avoid misdiagnosis and unnecessary interventions.
